# The Human African Trypanosomiasis Control and Surveillance Programme of the World Health Organization 2000–2009: The Way Forward

**DOI:** 10.1371/journal.pntd.0001007

**Published:** 2011-02-22

**Authors:** Pere P. Simarro, Abdoulaye Diarra, Jose A. Ruiz Postigo, José R. Franco, Jean G. Jannin

**Affiliations:** 1 World Health Organization, Control of Neglected Tropical Diseases, Innovative and Intensified Disease Management, Geneva, Switzerland; 2 World Health Organization, Regional Office for Africa, Brazzaville, Congo; 3 World Health Organization, Regional Office for the Eastern Mediterranean, Cairo, Egypt; Yale School of Public Health, United States of America

## Background

One century ago human African trypanosomiasis (HAT), also known as sleeping sickness, was believed to curb the development of colonial territories. As soon as the cause of the disease was clearly identified, colonial authorities established extensive control operations, fearing an unpopulated continent and a shortage of human labour to exploit natural resources.

Systematic screening, treatment, and patient follow-up was established in western and central Africa for the *gambiense* form of the disease while, animal reservoir and vector control was mainly implemented in eastern and southern Africa for the *rhodesiense* form.

By the 1960s, transmission was practically interrupted in all endemic areas, providing evidence that the elimination of the disease as a public health problem was feasible and could be achieved with basic tools. Thereafter, the rarity of cases led to a loss of interest in sustained surveillance, and the risk of re-emergence of the disease was overlooked. Thus in the 1980s the disease re-emerged. By the 1990s, flare-ups were observed throughout past endemic areas, leading to a worrisome increase in the number of reported cases. At this time, nongovernmental organizations (NGOs) played a crucial role in the control of HAT. However, their interventions were mainly focused on remote and insecure areas. As emergency operators, their policy understandably excluded support to National Sleeping Sickness Control Programmes (NSSCPs), which resulted in (i) the establishment of substitute HAT control systems (ii), the maintenance of a large part of the population at risk out of the umbrella of NGO projects, and (iii) the difficulty for national programmes to sustain control achievements after the NGOs' withdrawal. Concurrently, bilateral cooperation continued to support NSSCPs in some historically linked countries.

Concerning HAT screening, the card agglutination trypanosomiasis test (CATT) for serological screening of populations at risk of HAT *gambiense* was developed during the 1970s [1], but its large-scale production encountered many problems, hindering its availability [2]; in addition, production of anti-trypanosomal drugs was seriously threatened due to the lower economic return for manufacturers.

Research for new diagnostic tools and drugs was scarce [3]. Only eflornithine, initially developed for cancer treatment, was finally registered for the treatment of the *gambiense* form of the disease in 1990 [4]. But its cost and complex distribution and administration requirements made it inappropriate for the under-equipped peripheral health services in remote rural areas where HAT was prevalent. Only some well-funded NGOs were able to afford the cost of eflornithine treatment.

During the 1990s, security constraints due to civil wars and social upheavals complicated HAT control by preventing access to a large number of HAT-endemic areas, leading to difficulties in reaching a large number of affected populations and consequently to a considerable lack of epidemiological information. The World Health Organization (WHO) Expert Committee on HAT Control and Surveillance held in 1995, in consideration of the huge uncertainties between the reported cases and the factual field situation, estimated that the true number of cases was at least 10 times more than reported. Thus from the 30,000 reported cases annually, it was estimated that some 300,000 infected individuals remained ignored in the field [5].

In 1997, the 50th World Health Assembly expressed its concerns about the major recrudescence of cases by adopting a resolution to raise awareness and national and international interest [6].

Subsequently, WHO enhanced its coordinating role and promoted networking with partners, developing a strong advocacy and awareness campaign. As a result, the private sector recognized its responsibility, which led Aventis Pharma and Bayer Health Care to grant in 2001 and 2002 a substantial support to WHO for the control and surveillance of HAT. This support included HAT drug donation and financial contributions that allowed WHO to strengthen its support to disease-endemic countries (DECs).

The importance of the various components of the epidemiology of trypanosomiasis (human, animal, vector control, agricultural activity, and livestock production) and their impact on the development of rural Africa led WHO, in 1995, to promote together with the Food and Agriculture Organization (FAO), the International Atomic Energy Agency (IAEA), and the African Union InterAfrican Bureau for Animal Resources (AU-IBAR), an inter-sectoral initiative that ultimately became, in 1997, the Programme Against African Trypanosomiasis (PAAT, http://www.fao.org/ag/againfo/programmes/en/paat/disease.html).

In parallel, African heads of state and governments established during the African Union Summit in Lomé in 2000 the Pan African Tsetse and Trypanosomiasis Eradication Campaign (PATTEC, http://www.africa-union.org/Structure_of_the_Commission/depPattec.htm) with the objective to render Africa a tsetse- and trypanosomiasis-free continent.

## Current Situation

Between 2000 and 2009, out of 36 countries listed as endemic, 24 received the exclusive support of WHO either to assess the epidemiological status of HAT or to establish control and surveillance activities (Benin, Burkina Faso, Cameroon, Chad, Côte d'Ivoire, Gabon, Ghana, Guinea, Guinea Bissau, Kenya, Liberia, Malawi, Mali, Mozambique, Nigeria, Rwanda, Senegal, Sierra Leone, Swaziland, Togo, Uganda, United Republic of Tanzania, Zambia, and Zimbabwe); six received support from WHO as well as NGOs or through bilateral cooperation (Angola, Central African Republic [CAR], Congo, Democratic Republic of the Congo [DRC], Equatorial Guinea, and Sudan); and finally, six countries, Botswana, Burundi, Ethiopia, Gambia, Namibia, and Niger, which are listed as endemic but not having reported any cases in the last 20 years, have not received any support yet.

The 30 countries mentioned above received WHO support in the form of

- Technical assistance. It is provided either by WHO staff or by WHO temporary advisers.
- Access to diagnosis. This support includes the equipment, reagents, logistics, and funds to allow the national teams to reach HAT transmission areas to perform active case-finding surveys and set up passive surveillance.
- Training. As part of capacity building, targeted at two technical levels; (a) training on site, hands on (410 technical staff from 23 disease-endemic countries were trained); (b) participation in the International Course on African Trypanosomoses implemented in collaboration with the Association against Trypanosomiasis in Africa (105 programme managers or scientists from 22 countries have participated in either one of the five courses).
- Access to treatment. This covers the provision of drugs as well as patient accessibility. During the last decade, WHO has covered the need of DECs by distributing, in collaboration with Médecins sans Frontières (MSF)-Logistics, 594,200 vials of melarsoprol, 576,375 vials of pentamidine, 477,542 vials of eflornithine, and 13,597 vials of suramin.

**Figure 1:**
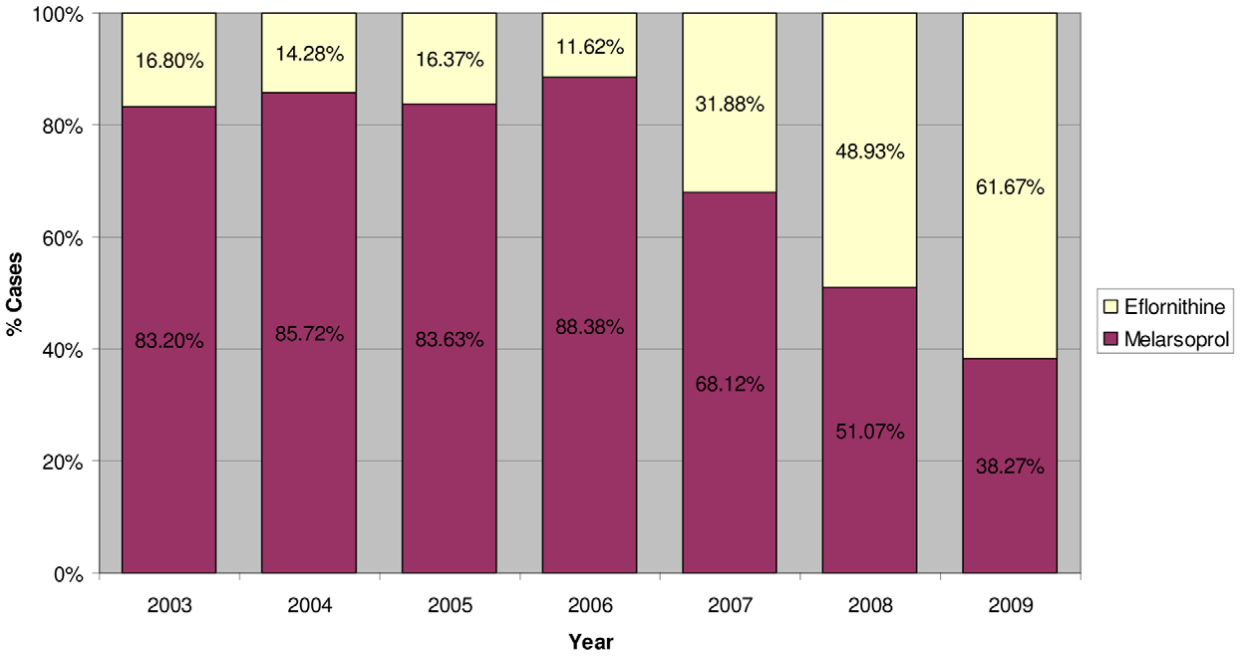
Percentage of second stage *T. b. gambiense* patients treated according to drug used. Eflornithine versus melarsoprol (2003–2009).

**Figure 2:**
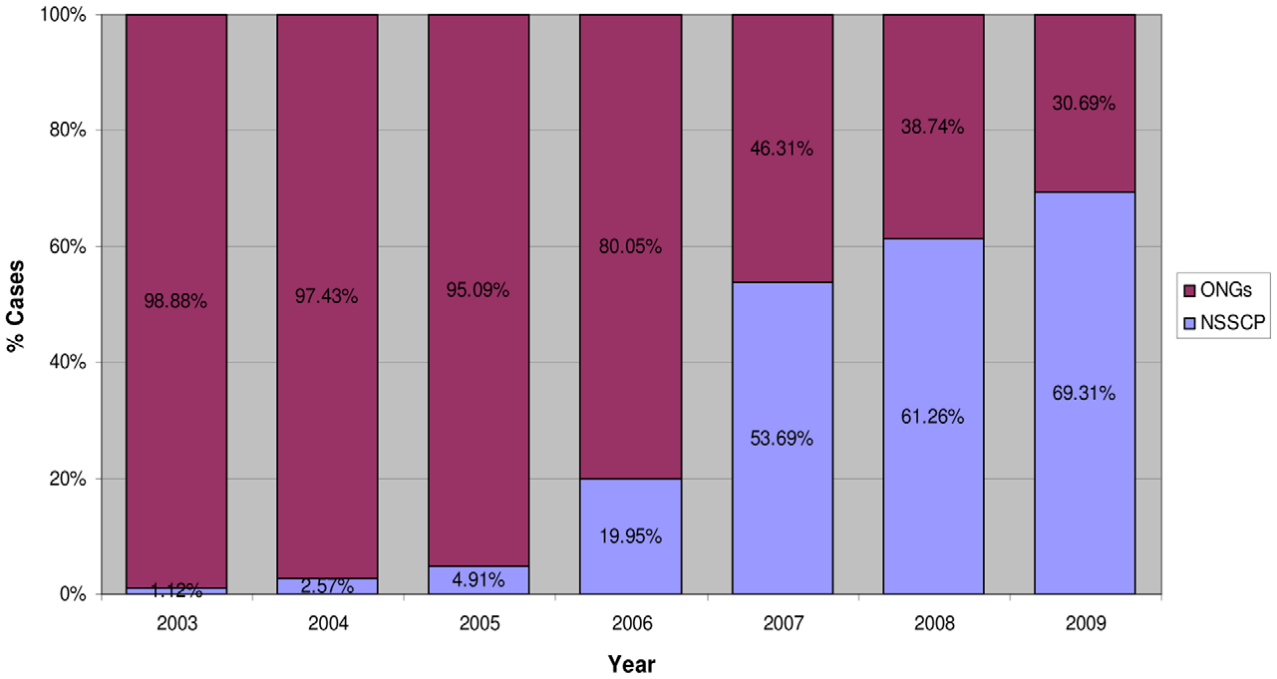
Institutional rate use of eflornithine. National Sleeping Sickness Control Programmes versus nongovernmental organizations (2003–2009).

One main objective of WHO in the “access to treatment” initiative was to reduce the use of the arsenic derivative melarsoprol for the treatment of second stage *gambiense* cases by making eflornithine, actually the sole alternative to melarsoprol, accessible. Indeed, during the period 2003–2006, despite the availability of eflornithine and the known toxicity of melarsoprol, the latter remained widely used and 88% of the second stage *gambiense* cases were treated with this drug (Figure 1). Only well-funded NGOs could afford the costly and complex use of eflornithine as first line treatment, while NSSCPs used eflornithine exclusively to treat melarsoprol relapses. This was demonstrated during the period 2003–2006 by a ratio of eflornithine distribution of 9 to 1 to NGOs versus NSSCPs (Figure 2).

In 2006, a number of DECs requested the support of WHO to train their staff on the use of eflornithine and requested the provision of the necessary equipment to switch gradually from melarsoprol to eflornithine as first line treatment. Subsequently, a training of trainers was organized in Southern Sudan and a kit containing the drugs as well as all the materials needed to administer two full eflornithine treatments was designed by WHO and distributed with the collaboration of MSF-Logistics [7]. The kit for two eflornithine treatments weighted 40 kg at a cost of US$1,420. This particular effort in terms of logistics and funding allowed DECs to regularly decrease their use of melarsoprol and increase the use of eflornithine for the treatment of second stage *gambiense* cases. Consequently, in 2009, a 57% reduction in the use of melarsoprol was recorded. Indeed, the percentage of patients treated with this drug fell from 88% to 38% (Figure 1), and subsequently the use of eflornithine by NSSCPs versus NGOs increased by 250% (from 20% to 70%) (Figure 2).

Nifurtimox, registered for Chagas disease, showed efficacy during compassionate use in melarsoprol refractory cases [8], [9]. In order to simplify the eflornithine schedule, attempts were made to demonstrate that a therapy combining nifurtimox and eflornithine could contribute to a simpler administration of the drugs; some trials took place in DRC during the late 1990s [10] and in Uganda during the early 2000s [11], [12].

In 2003, an extensive nifurtimox/eflornithine combination treatment (NECT) clinical trial started in Congo and later in DRC involving MSF, Epicentre, the Special Programme for Research & Training in Tropical Diseases (TDR), and Drugs for Neglected Diseases *initiative* (DND*i*). The trial ended in 2008. Results indicated that NECT presented no inferior efficacy and safety than the eflornithine monotherapy [13].

Following the inclusion of the NECT on the WHO Essential Medicines List in May 2009 [14], NSSCPs requested WHO to train their staff in order to incorporate this new combination in their national policy. A training for trainers was organized in Kinshasa in November 2009 for French speaking countries and another for English speaking countries in Uganda in February 2010 [15].

Thereafter, a new kit for NECT treatment was designed. Thanks to the reduction of drug quantity and materials, using the same packaging form as for the eflornithine monotherapy treatment kits, a new kit for four full NECT treatments weighting 36 kg at a cost of US$1,440 was produced. This kit has already been distributed to nine countries (reporting together 96% of all *Trypanosoma brucei gambiense* cases in 2009): Cameroon, CAR, Chad, Côte d'Ivoire, DRC, Equatorial Guinea, Gabon, Sudan, and Uganda.

However, NECT does not change the paradigm of HAT treatment since it remains logistically complicated to implement. Nevertheless, it is anticipated that NECT will contribute to sustain the already observed decreasing trend of melarsoprol use for the treatment of second stage *T. b. gambiense* infections [16].

During the period 2006–2009, WHO promoted research for better knowledge of HAT epidemiology and for the development of new tools. With that objective in mind, 23 agreements for “performance of work” were concluded with research institutions of 11 countries (Belgium, Burkina Faso, Democratic Republic of the Congo, France, Germany, Italy, Kenya, Malawi, Uganda, United Kingdom and the United Republic of Tanzania).

In 2006, WHO and the Foundation for Innovative New Diagnostics (FIND, http://www.finddiagnostics.org/) signed a 5-year Memorandum of Understanding to promote the development of simple and more sensitive and specific diagnostic tests. WHO took the responsibility to set up a specimen bank to facilitate the evaluation of relevant new diagnostic tools and to reduce the need for field trials. Currently, samples from 1,700 people including patients, seropositive-suspects, and controls have been collected from 14 sites in DRC, Guinea, Chad, Uganda, Malawi, and United Republic of Tanzania. More than 20,000 samples (including serum, plasma, white blood cells, urine, saliva, and CSF) are stored in the central repository bank at the Institut Pasteur in Paris.

Strong collaboration has been established with groups working on the development of new drugs, mainly the Consortium for Parasitic Drug Development (CPDD, http://www.unc.edu/~jonessk/) and DND*i* (http://www.dndi.org/).

In addition, the Division of Parasitic Diseases of the National Center for Infectious Diseases, Centers for Disease Control and Prevention in Atlanta, United States, the Parasite Diagnostics Unit from the Institute of Tropical Medicine (ITM) in Antwerp, Belgium, and the Research Unit of the Institut de Recherche pour le Développement (IRD) based in the International Centre for Research and Development in Livestock in Sub Humid Areas in Bobo-Diulaso, Burkina Faso (CIRDES), have been nominated as WHO Collaborating Centres.

In February 2008, WHO launched the Atlas of HAT initiative to map all reported cases for the period 2000–2009 at the village level. This initiative is jointly implemented with FAO in the framework of the PAAT. Presently, mapping includes 23 out the 25 countries having reported at least one case in the last 10 years. In the two remaining countries, Angola and DRC, data processing is ongoing. The Atlas database also includes epidemiological information that can be used by NSSCPs, NGOs, and research institutions to monitor and evaluate the impact of control activities, to assess epidemiological trends, and to plan control or research activities [17].

**Figure 3:**
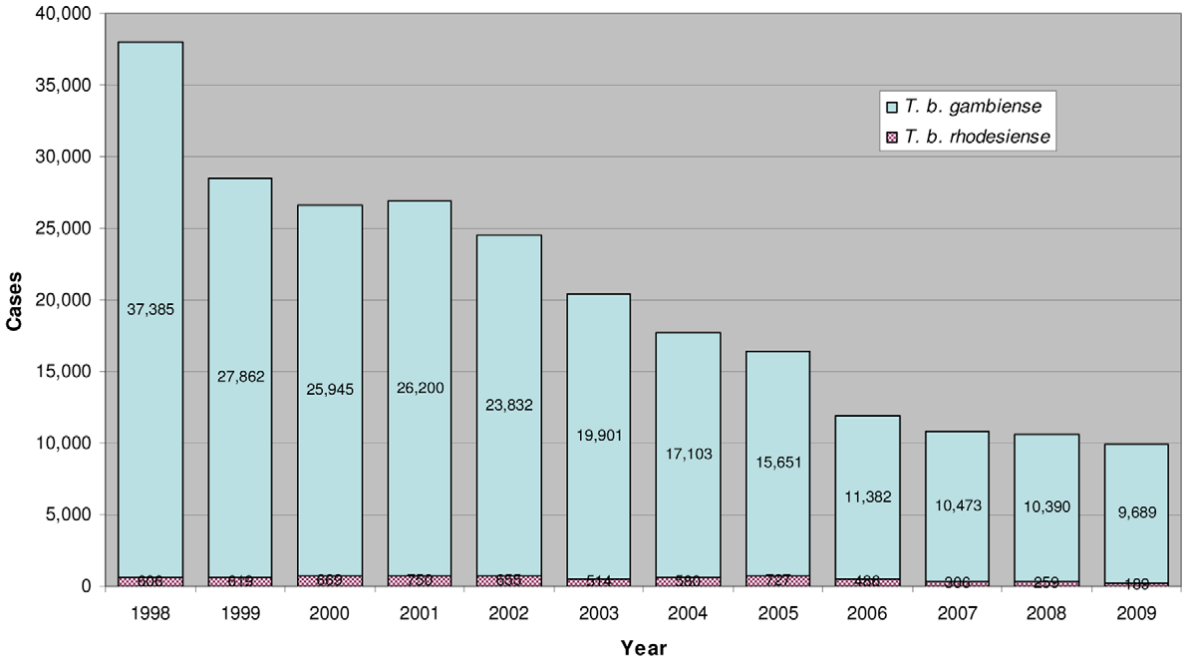
Evolution of reported cases of both forms of human African trypanosomiasis (1998–2009).

As a consequence of these activities, the number of new cases reported to WHO in 2009 has dropped below 10,000 for the first time in 50 years [18]. It represents a decrease of 63% since 2000 (Figure 3). In 2009, only two countries have reported more than 1,000 new cases, namely CAR and DRC representing, respectively, 11% and 73% of the total cases reported. One country, Chad, has reported more than 500 but less than 1,000 new cases. Three countries (Angola, Sudan, and Uganda) have reported more than 100 but less than 500 new cases. Eleven countries have reported less than 100 cases: Cameroon, Congo, Côte d'Ivoire, Equatorial Guinea, Gabon, Guinea, Kenya, Malawi, United Republic of Tanzania, Zambia, and Zimbabwe.

**Figure 4:**
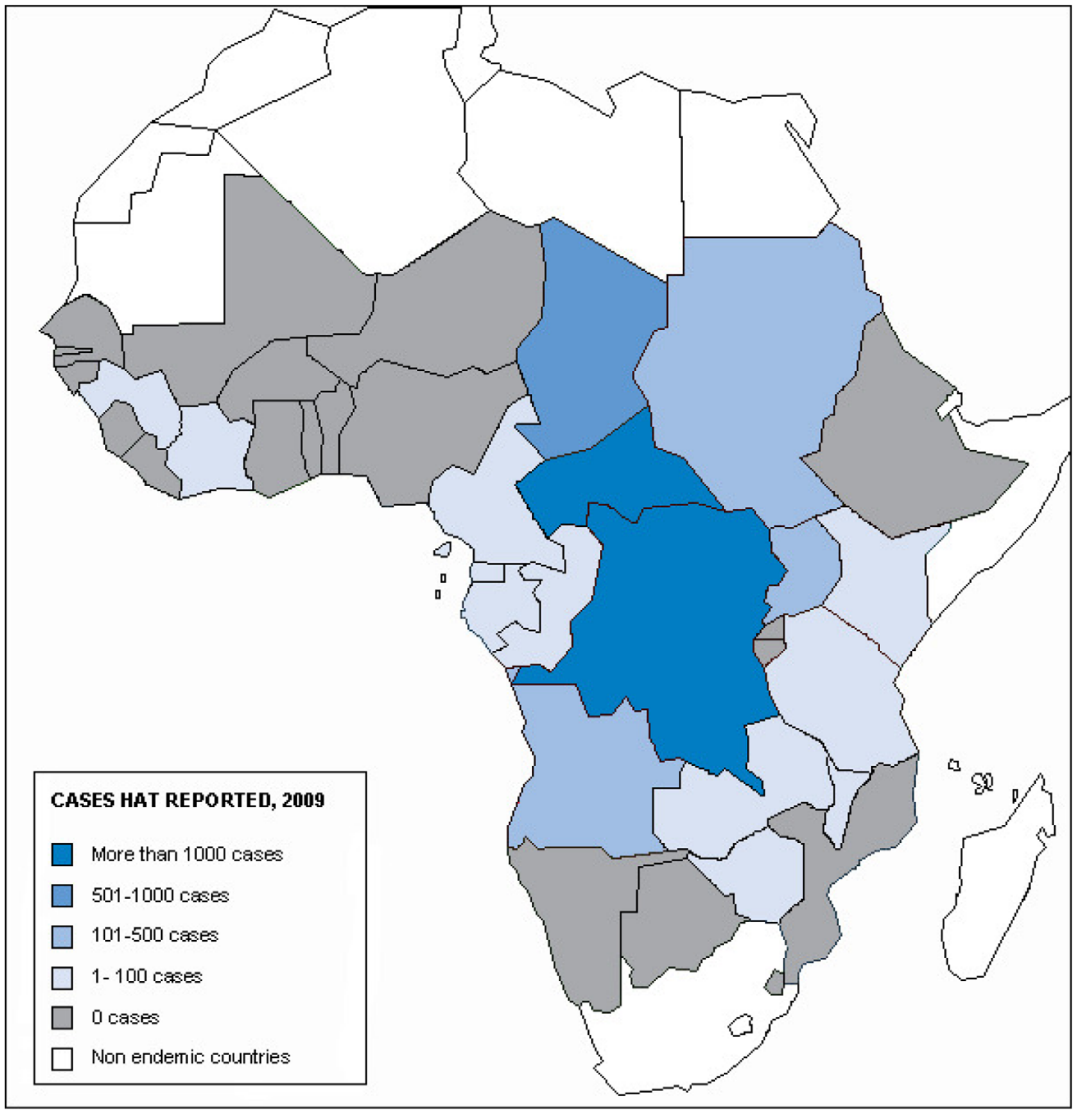
Classification of human African trypanosomiasis-endemic countries according to cases reported in 2009.

Finally, 19 countries listed as being HAT endemic reported no cases in 2009. Seven of these have performed HAT surveillance activities: Benin, Burkina Faso, Ghana, Mali, Nigeria, Sierra Leone, and Togo. Nine have no regular surveillance activities but have reported no cases for decades. These include Burundi, Ethiopia, Gambia, Guinea Bissau, Liberia, Mozambique, Niger, Rwanda, and Senegal; however, these latter countries deserve an assessment to clarify their epidemiological situation. Two countries, namely Botswana and Namibia, are considered disease transmission free due to the recently implemented, successful tsetse elimination campaigns [19], [20]. Finally, Swaziland has been shown through an extensive tsetse survey to harbour *Glossina austeni*, which has been never described as a HAT vector [21] (Figure 4).

## Discussion

During the last decade, the WHO public private partnership (PPP) established in 2001 with Aventis Pharma and renewed in 2006 by sanofi-aventis has made possible to carry out extensive HAT control activities and to strengthen the capacities of NSSCPs. The PPP has been complemented by bilateral cooperation, NGOs, research institutes, and Bayer AG's support. Furthermore, the cessation of civil wars and social upheavals has also substantially facilitated access to HAT-endemic areas.

In 2009, the number of new cases of HAT reported to WHO has dropped below the symbolic number of 10,000, while in the period 2000–2009 the number of people screened increased due to the greater number of health care facilities involved in passive screening and the improvement of the performance of active case-finding surveys. Due to the improved knowledge of HAT distribution, WHO estimated in 2006 the factor gap between cases reported and cases infected to be three [22] instead of ten, as was thought in 1995 [5].

Considering the next steps to be implemented, it is important to note that the disease situation is not homogeneous throughout the continent.

The *gambiense* form of the disease has in several foci already reached a prevalence threshold compatible with the concept “eliminated as a public health problem”. To consolidate such results, and ensure sustainability, an adapted control and surveillance approach will have to be implemented within the national health system. Whereas in other foci HAT remains a public health issue, it is mostly due to accessibility problems or security constraints [23]. Therefore, reinforced control measures must be maintained using the classical vertical approaches with the participation of existing health care structures.

The *rhodesiense* form of HAT is a zoonotic disease involving cattle and game in the transmission cycle. Cattle movement is a continuous threat of disease transmission as well as spread and subsequently a source of outbreaks [24]. Furthermore, wildlife in protected areas are niches for contamination; there is a continuous risk for park rangers, the surrounding population, and visitors to become infected. Controlling this form of the disease requires a multisectoral approach. Therefore, it is crucial to reinforce local health care capacities for diagnosis and disease management as well as to establish effective coordination with veterinary and natural resources management services in charge of domestic animals, wild animals, and vector control.

Despite encouraging results and exciting perspectives, the process remains fragile. At this stage, some obstacles are anticipated in the course of future control activities and a few issues should be carefully considered. These are mainly:

- The decline on contribution by NGOs and bilateral cooperation towards HAT control. During the period 2000–2009 there were nine bilateral and 38 NGO HAT projects, while in 2010 there remained only one bilateral (DRC) and five NGO projects (CAR, DRC, Sudan, and Uganda). The positive aspect of this situation is the decrease in HAT-related emergencies and the substantial improvement of country self-managed HAT control activities.
- The "tyranny of disability adjusted life years (DALYs)” expresses the lack of interest of donors when the burden of the disease is decreasing. Then, supporting institutions not only withdraw from HAT control but also from HAT research. With the reduced amount of funds available for control, it seems obvious that the responsibility to give “the last strike to the dying beast” will exclusively rely on the overloaded and weak national health services. Also, the loss of support for research will definitely eliminate any hope to get the needed, so long awaited new tools, not only to accelerate the current control process but also to boost the involvement of health services in HAT surveillance and control in order to sustain the achieved results. Such a situation will likely open the door for the re-emergence of the disease.
- While the control of cattle as a HAT reservoir appears to be a reachable objective that would in turn allow the control of *T. b. rhodesiense* infections in affected areas [25], the control of the disease in wildlife and the vector in protected areas and game reserves could be more complicated due to conservationist, ecological, and environmental considerations.

Furthermore, close monitoring is needed to assess the impact of climate changes and demographic evolution [26], [27] in HAT transmission.

## Conclusion

By the end of the last century, WHO and its partners had developed a strong and successful advocacy programme to secure access to diagnosis and treatment, ensuring availability of funds and drugs to support DECs. As a result, during the first decade of the current century, great advances have been made in HAT control.

In 2007, a WHO informal consultation of the heads of NSSCPs held in Geneva reached the conclusion that elimination of the disease as a public health problem was possible [28]. This conclusion was based on the achievements obtained, on the current understanding of the epidemiology of the disease, and on the willingness of African heads of states and governments to eradicate tsetse and trypanosomiasis as stated when the PATTEC was established in 2000.

The time has now come to sensitize stakeholders on the pertinence and ethical duty of embarking on the process of eliminating HAT as a public health problem despite the difficulties, obstacles, and threats that are expected in this process. Without such hammering approach, there is a risk of stagnation in control and surveillance as occurred in the late 1960s that ultimately led to the return of the disease.

Today, WHO and its partners are committed to reinforcing and coordinating actions towards a sustainable elimination process [29]. While there are still technical aspects to be solved, the elimination of HAT as a public health problem will require social peace, institutional support, and adequate funding for its implementation. These last conditions are not exclusive to the control, elimination, and sustained surveillance of HAT but also for the overall development of DECs, which would contribute to the control of HAT as well.

When targeting the elimination of HAT as a public health problem, the goal should be recognized as a major achievement but must never be considered as an end point. Without appropriate discrimination, the use of the word “elimination” may lead to risky conclusions. The disease believed to “no longer exist” will reach oblivion, placing in the background the required pressing efforts for a sustained and effective surveillance. It must be kept in mind that "elimination" is not synonymous with “eradication”. Elimination is only a point in time in the control process of the disease, at which stage the classical vertical control intervention approaches are no longer cost effective. Thus, the national health system must take the ownership of sustaining elimination by integrating HAT surveillance in their services while maintaining the capacity to react rapidly according to the analytical results of the surveillance outcome.

Elimination should be considered as the beginning of a new process involving new actors. Therefore, elimination of HAT as a public health problem will require continuous efforts and innovative approaches. There is no doubt that new tools would facilitate the elimination process and the sustainability of results; thus, funding efforts for HAT control and research must continue based on public health objectives, and no longer on the burden of the disease.
